# Influenza and Respiratory Virus Surveillance, Vaccine Uptake, and Effectiveness at a Time of Cocirculating COVID-19: Protocol for the English Primary Care Sentinel System for 2020-2021

**DOI:** 10.2196/24341

**Published:** 2021-02-19

**Authors:** Simon de Lusignan, Jamie Lopez Bernal, Rachel Byford, Gayatri Amirthalingam, Filipa Ferreira, Oluwafunmi Akinyemi, Nick Andrews, Helen Campbell, Gavin Dabrera, Alexandra Deeks, Alex J Elliot, Else Krajenbrink, Harshana Liyanage, Dylan McGagh, Cecilia Okusi, Vaishnavi Parimalanathan, Mary Ramsay, Gillian Smith, Manasa Tripathy, John Williams, William Victor, Maria Zambon, Gary Howsam, Brian David Nicholson, Victoria Tzortziou Brown, Christopher C Butler, Mark Joy, FD Richard Hobbs

**Affiliations:** 1 Nuffield Department of Primary Care Health Sciences University of Oxford Oxford United Kingdom; 2 Public Health England London United Kingdom; 3 Royal College of General Practitioners London United Kingdom

**Keywords:** COVID-19, general practice, influenza, computerized medical record systems, sentinel surveillance, coronavirus infections, records as topic, serology, virology

## Abstract

**Background:**

The Oxford–Royal College of General Practitioners (RCGP) Research and Surveillance Centre (RSC) and Public Health England (PHE) are commencing their 54th season of collaboration at a time when SARS-CoV-2 infections are likely to be cocirculating with the usual winter infections.

**Objective:**

The aim of this study is to conduct surveillance of influenza and other monitored respiratory conditions and to report on vaccine uptake and effectiveness using nationally representative surveillance data extracted from primary care computerized medical records systems. We also aim to have general practices collect virology and serology specimens and to participate in trials and other interventional research.

**Methods:**

The RCGP RSC network comprises over 1700 general practices in England and Wales. We will extract pseudonymized data twice weekly and are migrating to a system of daily extracts. First, we will collect pseudonymized, routine, coded clinical data for the surveillance of monitored and unexpected conditions; data on vaccine exposure and adverse events of interest; and data on approved research study outcomes. Second, we will provide dashboards to give general practices feedback about levels of care and data quality, as compared to other network practices. We will focus on collecting data on influenza-like illness, upper and lower respiratory tract infections, and suspected COVID-19. Third, approximately 300 practices will participate in the 2020-2021 virology and serology surveillance; this will include responsive surveillance and long-term follow-up of previous SARS-CoV-2 infections. Fourth, member practices will be able to recruit volunteer patients to trials, including early interventions to improve COVID-19 outcomes and point-of-care testing. Lastly, the legal basis for our surveillance with PHE is Regulation 3 of the Health Service (Control of Patient Information) Regulations 2002; other studies require appropriate ethical approval.

**Results:**

The RCGP RSC network has tripled in size; there were previously 100 virology practices and 500 practices overall in the network and we now have 322 and 1724, respectively. The Oxford–RCGP Clinical Informatics Digital Hub (ORCHID) secure networks enable the daily analysis of the extended network; currently, 1076 practices are uploaded. We are implementing a central swab distribution system for patients self-swabbing at home in addition to in-practice sampling. We have converted all our primary care coding to Systematized Nomenclature of Medicine Clinical Terms (SNOMED CT) coding. Throughout spring and summer 2020, the network has continued to collect specimens in preparation for the winter or for any second wave of COVID-19 cases. We have collected 5404 swabs and detected 623 cases of COVID-19 through extended virological sampling, and 19,341 samples have been collected for serology. This shows our preparedness for the winter season.

**Conclusions:**

The COVID-19 pandemic has been associated with a groundswell of general practices joining our network. It has also created a permissive environment in which we have developed the capacity and capability of the national primary care surveillance systems and our unique public health institute, the RCGP and University of Oxford collaboration.

## Introduction

### Background

The Oxford–Royal College of General Practitioners (RCGP) Research and Surveillance Centre (RSC) is a network of general practices and is now in its 54th season of surveillance and vaccine effectiveness in its long-standing collaboration with Public Health England (PHE) and predecessor bodies [1]. This season is complicated by the likely cocirculation of SARS-CoV-2 and its associated disease burden [2].

The RCGP RSC has been recruited as a nationally representative population [3] that provides pseudonymized data for surveillance of infectious diseases. However, the recent influx of practices may mean we have to adjust for practice distribution and ensure we recruit virology sampling practices that are evenly distributed. The disease surveillance program is commissioned by PHE and monitors 38 infectious diseases, including influenza; at the start of 2020, monitoring extended to include COVID-19 [4].

The recently expanded RCGP RSC extracts pseudonymized data from over 1700 general practices each week covering a nationally representative [3] population of over 13 million. Data from these practices are reported online in the RCGP’s Weekly Return [5], which includes monitoring weekly rates of influenza-like illness (ILI) and other communicable and respiratory diseases for England. We also produce an annual report [6]. The RCGP RSC data set includes all coded data and all prescribed items, including vaccine exposure [3]. To manage our extended network of practices, we have set up a new analytics hub: the Oxford–Royal College of General Practitioners Clinical Informatics Digital Hub (ORCHID) [7].

**Figure 1:**
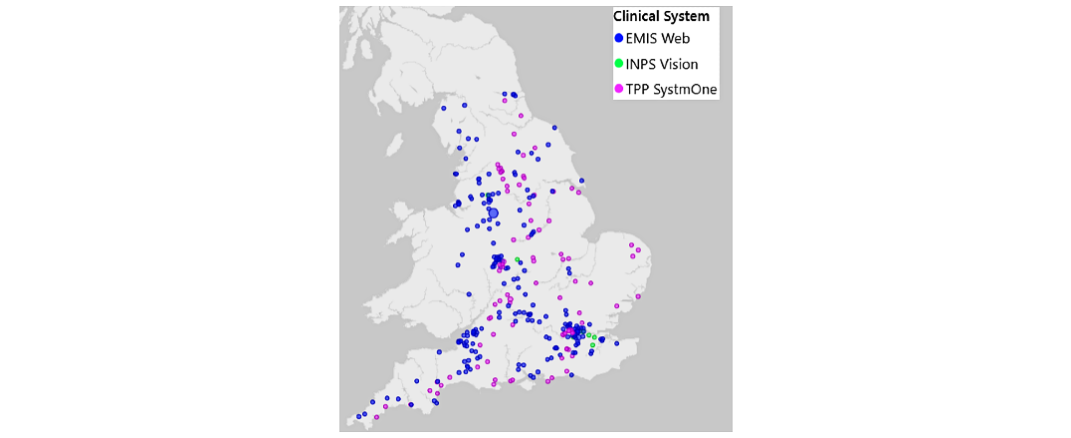
Royal College of General Practitioners Research and Surveillance Centre’s virology sampling sites in England. Distribution by type of computerized medical record system is shown. EMIS: Egton Medical Information Systems; INPS: In Practice Systems; TPP: The Phoenix Partnership.

The RCGP RSC conducts virology surveillance each influenza season. Given the onset of COVID-19, virological sampling has expanded to approximately 300 general practices (see Figure 1). We have reported on which groups of patients were more likely to test positive for COVID-19 [8], on excess mortality [9], and the shift away from face-to-face consultations during the first wave of COVID-19 pandemic [10].

The RCGP RSC is also active in serosurveillance. During the 2018-2019 influenza season, we successfully conducted our first collection of serological samples from adults, linking them to their respective medical records [11,12]. Sentinel networks can provide a mechanism for systematic data collection and linkage to medical records and health outcomes [13], as well as sampling across the complete age range [14]. Current activity has included supporting PHE household studies and collecting convalescent sera from people previously infected with COVID-19.

Finally, the RCGP RSC network has supported trials and studies designated as high-priority public health research. These included recruiting to the PRINCIPLE (Platform Randomised trial of INterventions against COVID-19 In older peoPLE) trial [15] and supporting recruitment to RAPTOR-C19 (RAPid community Testing fOR COVID-19) [16].

This protocol sets out how the RCGP RSC will continue to provide enhanced passive surveillance, virological and serological surveillance from a subset of practices, and support high-priority public health research.

### Aim

The aim of this protocol is to provide comprehensive surveillance of influenza, COVID-19, and other monitored conditions including health outcomes. The RCGP RSC network will also collect samples for virological and serological analysis, measure vaccine exposure and effectiveness, and support other high-priority public health research.

### Objectives

Our six objectives are as follows:

1. To conduct surveillance of influenza and other monitored respiratory conditions, including COVID-19. This will be primarily from primary care data but will also include linkage to secondary care and mortality data sets to enable reporting of health outcomes. We will provide observatories, dashboards, and training to optimize data quality.
2. To collect virological and serological samples to report the presence and spread of monitored conditions from a nationally representative sample of practices.
3. To report vaccine uptake, estimate effectiveness, and investigate adverse events of interest (AEIs). We will focus on influenza vaccinations and possible new COVID-19 vaccines.
4. To support research to answer important public health questions, including trials of early interventions in the community, near-patient testing, and household spread.
5. Apply the Findable, Accessible, Interoperable, and Reusable (FAIR) principles so that our data are findable, accessible, interoperable, and reusable by other organizations.
6. To implement appropriate information and research governance.

## Methods

### Overview

The methods include six components: (1) surveillance of influenza, COVID-19, other monitored conditions, and associated health outcomes; (2) virological surveillance and serosurveillance; (3) monitoring vaccine uptake and effectiveness; (4) supporting priority public health research; (5) data curation; and (6) governance.

### Surveillance of Influenza, COVID-19, Other Monitored Conditions, and Associated Health Outcomes

We will collect pseudonymized, routinely collected, coded clinical data, which has been coded into patient records, using UK Systematized Nomenclature of Medicine Clinical Terms (SNOMED CT) codes (SNOMED International), for enhanced passive surveillance. This will entail a wide range of clinical event data, including collection of monitored conditions, vaccine exposure, AEIs, hospital admissions, intensive care, and deaths, to monitor approved study outcomes and for any unexpected public health emergencies. Effective interoperability and communication will be facilitated by monthly pseudonymized linkage, facilitated by NHS (National Health Service) Digital and PHE [7], to secondary care and other data sources, including Secondary Uses Service, the continuously updated source of hospital data (see Textbox 1).

**Linkage to other data sets to support surveillance and research; individual data sets may have limitations on their use to specific projects and users.**

**NHS (National Health Service) Digital:**

- Cancer Registration Data (reduced)
- Secondary Uses Service (SUS) Payment by Results Episodes
- SUS Payment by Results Outpatients
- SUS Payment by Results Accident and Emergency
- SUS Payment by Results Spells
- Mental Health Services Data Set
- Diagnostic Imaging Dataset
- Emergency Care Data Set (ECDS)
- COVID-19 Hospitalisation in England Surveillance System (CHESS) Dataset-CV19 NHS 111 Online
- Second Generation Surveillance System (SGSS): laboratory test reports
- Hospital Episode Statistics (HES; same granularity of supply as for SUS)
- Office of National Statistics (ONS) Mortality
- NHS 111 (free-to-call single nonemergency number medical helpline)

**National Cancer Registration and Analysis Service (NCRAS):**

- Cancer Registry (full)
- Chemotherapy data set
- Radiotherapy data set

We have converted all our primary care coding to SNOMED CT coding and are using this throughout our data processing [17] (see Multimedia Appendix 1). We have been at the forefront of ensuring that primary care systems code cases correctly and that we comprehensively capture historic data [18]. This has included the development of database-linked tools to programmatically search SNOMED CT for relevant tools. The hierarchical nature of SNOMED CT is that it requires machine searching rather than manual searching (see Multimedia Appendix 2). This will continue to deliver effective COVID-19 surveillance in parallel with the annual influenza surveillance this season.

We will enhance surveillance through the provision of dashboards and observatories to provide feedback about levels of care compared to other practices in our network and to improve data quality, clinical care, and patient safety. There will be a particular focus on data quality, including the recording of ILIs, the use of the RCGP RSC case definition (see Textbox 2 [19-21]), recording of upper and lower respiratory tract infections, and recording of suspected COVID-19 cases, with an emphasis on recording episode type (ie, whether this is a new case or a follow-up). We will programmatically adjust for when episode type is not recorded [18,22].

**Figure 2:**
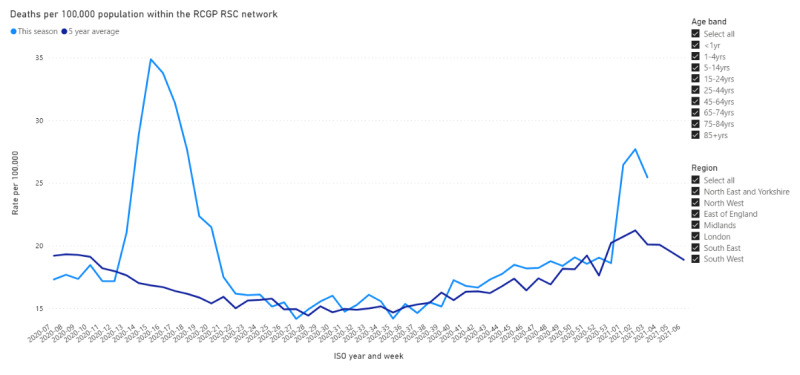
Royal College of General Practitioners Research and Surveillance Centre observatory showing mortality through the first wave of the COVID-19 pandemic in England. Deaths per 100,000 population are displayed over the International Organization for Standardization (ISO) weeks for 2019 and 2020.

This year we will extend our virology sampling window to 10 days. We will also report on health outcomes, including mortality [9] (see Figure 2).

**Royal College of General Practitioners (RCGP) Research and Surveillance Centre (RSC) case definitions of influenza-like illness (ILI).**

**The RCGP RSC definition of ILI:**

- An acute respiratory illness with a temperature measured as, reported as, or plausibly ≥38 °C and cough, with onset within the past 10 days.  ILI cases should not have another more plausible diagnosis. ILI cases have a sudden onset, and there are often symptoms suggestive of systemic upset—myalgia, fatigue, malaise, headache, etc.  RCGP RSC stresses the notion of symptoms within 10 days of onset to differentiate acute episodes, with swabs only wanted within 7 days of onset.
- This definition is compatible with the World Health Organization (WHO) and European Centre for Disease Prevention and Control (ECDC) definitions [19,20]. The WHO definition has the highest specificity (21.4%) and the ECDC definition has the highest sensitivity (96.1%) [21].

### Virological Surveillance and Serosurveillance

A volunteer subset of practices will conduct virological surveillance (see Multimedia Appendix 3). They will conduct or arrange virological sampling of patients presenting with ILI or lower respiratory tract infection, including acute bronchitis and bronchiolitis in children under the age of 5 years. We will include those suspected to have, or have been exposed to, COVID-19 who have persistent cough, loss of taste and/or smell, shortness of breath, history of fever, and/or the presence of a wheeze. We will look for practices to collect 20 samples per week; additional samples can be collected if the spread that is present is within a household or a communal establishment. Practices will receive feedback via dashboards [23] (see Textbox 3 [5,24-28]). Our target is to collect 600 samples per week across all age bands.

**Figure 3:**
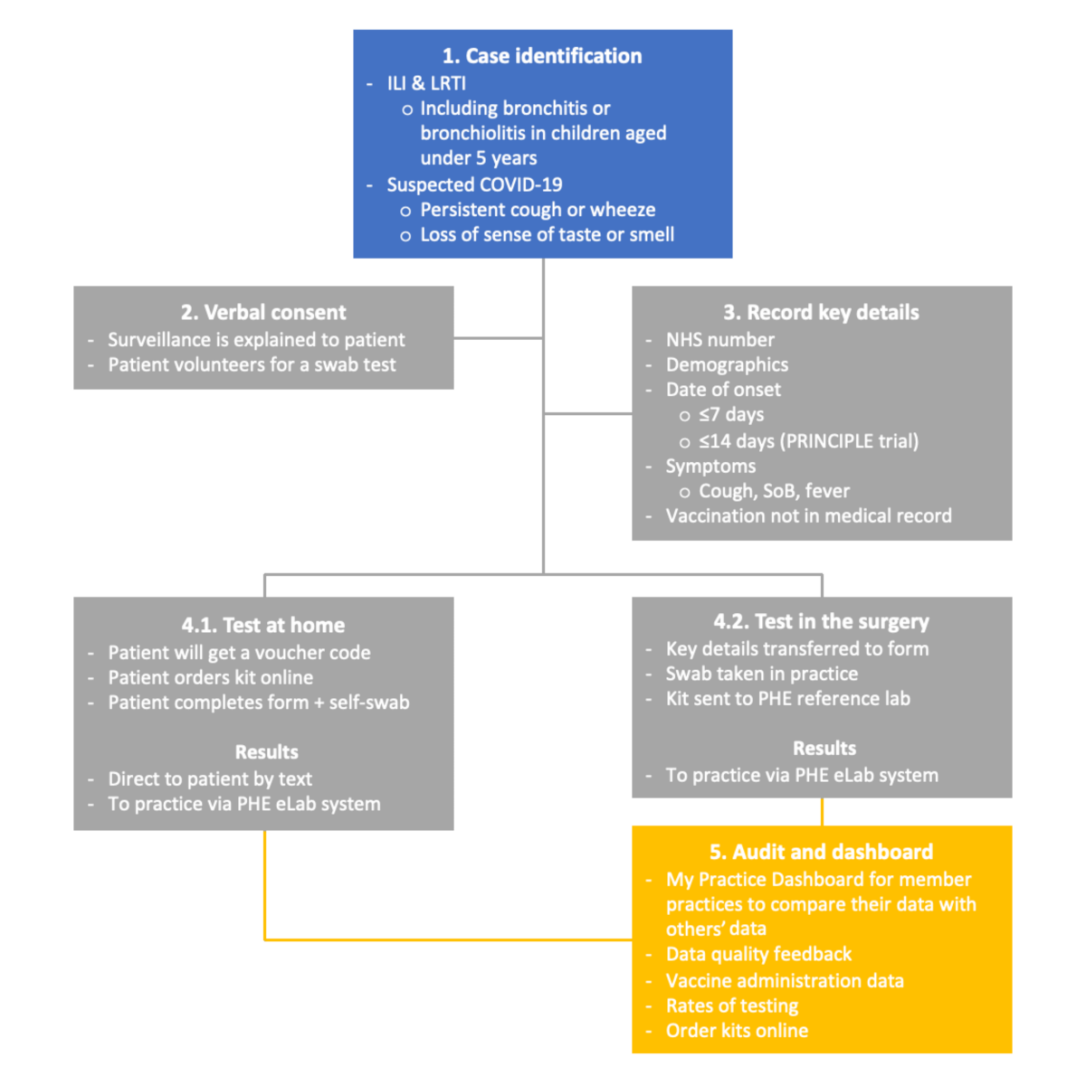
Flowchart for case identification and sampling for the 2020-2021 season surveillance. The National Health Service (NHS) number is a vital unique identifier for all forms across virology and serology, including for patients requesting a test kit at home. ILI: influenza-like illness; LRTI: lower respiratory tract infection; PHE: Public Health England; PRINCIPLE: Platform Randomised trial of INterventions against COVID-19 In older peoPLE; SoB: shortness of breath.

The anticipated changes in the coming year are as follows:

1. We will reduce the burden of information collected on the virology request form. Thus, a common but reduced set of data will be collected for all patients offered virology sampling. Vaccination history and a record of their comorbidities will have already been collected electronically as part of the standard data extract. However, we will stress the importance of recording the NHS number as a unique identifier, to help ensure efficient linkage of clinical data to test results.
2. People who volunteer to self-swab will be given a voucher code they can use online to arrange for a sample to be sent; those in the surgery will have a swab taken by their general practitioner or practice team, and this will be sent through the post to the PHE reference laboratory. Patients taking self-swabs should be given their NHS number to include in their swab request to improve data matching.
3. All results will be sent to practices online via the e-Lab system [29]; patients who self-swab and provide a mobile number will additionally receive their results by text message (see Figure 3).

**Dashboards, online support, and observatories—also called weekly returns—to monitor and support data recording and key public health projects.**

*Practice Dashboard*

My Practice Dashboard [24]

**All practices:**

- Seasonal wellness: all practices can see vaccine uptake and incidence of key monitored conditions
- COVID-19: all practices will compare their incidence of COVID-19 with the rest of the network

**Volunteer practices involved in specific surveillance or trials:**

- Virology: for practices participating in specimen collection; in-practice and home tests
- Serology: for practices to observe their serology sampling; primarily by age band
- PRINCIPLE (Platform Randomised trial of INterventions against COVID-19 In older peoPLE ) trial: to help practices know their level of recruitment
- Online support:
  Facebook page [25]
  Twitter account [26]
  Podcast [27]

*Observatories and Weekly Returns*

**Oxford–Royal College of General Practitioners (RCGP) Research and Surveillance Centre (RSC) weekly return:**

- Twice weekly report—during the pandemic—on influenza, COVID-19, and other monitored conditions; reports are published at the RCGP RSC website [5]—follow the “RSC Communicable and Respiratory Disease report” link on the “Public Health Data” page; this is the 54th year of this report

**Observatories [28]. We produce weekly reports online for the observatories, which provide an interactive weekly return of national data; these are as follows:**

- Seasonal wellness
- COVID-19
- Mortality
- Virology sampling
- Serology sampling
- PRINCIPLE trial eligible patients

**Figure 4:**
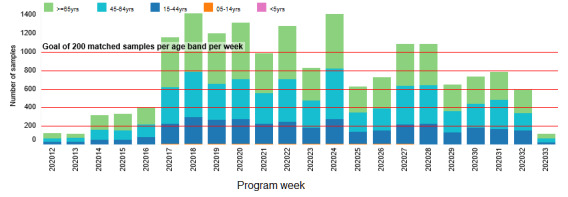
Number of serological surveillance samples collected per week. The program was initiated in week 11; we have been taking active steps to encourage similar numbers across age bands per week.

A volunteer group of practices will also participate in serological surveillance. The largest project will be the opportunistic collection of an extra blood sample from patients undergoing a routine blood test. Practices will be asked to collect across age bands (aged 10 years and above) (see Figure 4), with feedback provided to general practices via a dashboard and overall via an observatory [30]. We are now recruiting practices from areas with more ethnically mixed populations to provide greater insight into disease spread in ethnic minority populations. Other serology projects include extended serosurveillance in a subset of patients with positive virology tests (n=63) as well as recruiting and sampling patients included in the PHE-led household contacts of confirmed COVID-19 cases (HOCO) study [30]. We are piloting responsive surveillance in areas with higher disease incidence.

### Monitoring Vaccine Uptake, Effectiveness, and Adverse Events of Interest

Capturing vaccine exposure is critical to conducting vaccine effectiveness studies. With an increasing range of vaccines available, capturing exposure data when not recorded within the practice, beyond the fact that a vaccine has been given, presents significant challenges [31]. Further issues include difficulties capturing vaccine batch numbers [32]. We will ask practices to preload the vaccine brands and batch numbers online.

We plan to continue to report AEIs following vaccination using routinely collected data, though we would have the capability to provide patient AEI reporting cards if commissioned for COVID-19 or other vaccines [33,34]. Practices will be provided with prompts to improve coding. Good data quality regarding AEI recording may identify any differences between influenza vaccine types, to elucidate whether egg- or cell-based vaccines with or without adjuvants are associated with different rates of AEIs. There may also be up to six types of COVID-19 vaccines available for which we may be able to provide uptake and AEI data.

### Supporting Priority Public Health Research

#### Overview

The RCGP RSC will support a range of research (see Multimedia Appendix 4), with the PRINCIPLE [15] and RAPTOR-C19 [16] trials as the top priorities. The RCGP RSC is a member of a number of European studies, including a point-of-care testing study and data sharing as part of the DRIVE (Development of Robust and Innovative Vaccine Effectiveness) consortium [35,36] and a European study of respiratory syncytial virus epidemiology [37]. In partnership with PHE, we are also members of the I-MOVE (Influenza–Monitoring Vaccine Effectiveness in Europe) consortium, which has extended its work into COVID-19 [38].

We will support two ethically approved generic protocols for COVID-19 research: DECISION-COVID (DEfining the CharacterIStIcs Of Individuals with suspected Novel COronaVIrus Disease and risk factors for development of the disease) and MAINROUTE (Monitoring Attendance, INvestigation, Referral, and OUTcomEs in primary care: impact of and recovery from COVID-19 lockdown). DECISION-COVID provides a framework for looking at medications that might impact on COVID-19 outcomes; MAINROUTE assesses the impact of lockdown on the wider implications to care (see Multimedia Appendix 4). We have already reported on how lockdown has led to a reorganization of primary care service delivery [10]; in addition, we are part of the RECAP (Remote COVID-19 Assessment in Primary Care) early warning score validation study, which was set to start in September 2020 [39].

#### Statistical Methods

A range of statistical methods will be used that are appropriate to the area of surveillance or the research undertaken. We will use propensity matching and a wide range of other methods to work within the constraints of routine data [40].

Vaccine effectiveness will be assessed using test-negative design [41,42]. Patients meeting the predefined clinical criteria will undergo laboratory testing for specific circulating viruses. Patients who test positive will comprise the cases; patients who test negative to both influenza and COVID-19 will form the control group. In the clinical at-risk populations, as defined by NHS England, vaccine effectiveness will be calculated as follows: (1 − adjusted vaccination odds ratio) × 100% [42]. This will be estimated overall and stratified by influenza virus subtype for all ages. Cohort analysis will also be performed with vaccine effectiveness estimated as 1 – risk in vaccinated subjects and 1 – risk in unvaccinated subjects, with adjustment for confounding variables.

We will report AEIs using self-controlled case series design [43,44]. An exposure period will be identified shortly after vaccination, and a baseline period will form the remainder of the observation period. The frequency of AEIs will be compared to identify any invariant confounding [44,45]. We will use self-controlled case series to report the different rates of AEIs between vaccines; biases should be similar between vaccines. For influenza, we will continue to use the European Medicines Agency–specified AEIs; should COVID-19 vaccination commence this season, we will identify AEIs. Notwithstanding the enlarged size of the network, we are not large enough to detect rare events.

### Data Curation

We strongly support the FAIR principles [46]. We are a founding member of the Health Data Research UK Data Alliance, and our data are included in their metadata, with our activities ranked as high priority [47]. We are creating a data set within ORCHID, an observational research platform that has themed databases that can be made readily available to researchers simultaneously; these will be used to support DECISION-COVID and other studies (see Multimedia Appendix 4) [48]. Additionally, we are linking over 50 years of virology and RCGP RSC data, as part of the Wellcome Trust–funded quinquagenarian database project, to make these data more accessible [49]. ORCHID [7] aims to provide a large, near-real-time–themed primary care health informatics hub for the use of data from consenting patients in clinical trials and to supplement existing disease surveillance using in situ network data without large-scale data extraction. We aim to make outputs available using common data models, which we will be providing to the European Health Data Evidence Network [50,51].

### Governance

The surveillance for the coming year will be initiated by a joint letter to volunteer practices informing them of their role in the surveillance process. The legal basis for our surveillance with PHE is defined as Health Protection under Regulation 3 of the Health Service (Control of Patient Information) Regulations 2002 and is approved annually by the PHE Caldicott Guardian; other studies require appropriate ethical approval. The University of Oxford is compliant with the General Data Protection Regulation and the NHS Digital Data Security and Protection policy.

We want to minimize any risk associated with taking part in this surveillance. Infection prevention and control advice will follow extant national guidance. Our member practices already have most processes in place to meet Regulation 12 of the Health and Social Care Act 2008 (Regulated Activities) Regulations 2014, concerning safe care and treatment, and are periodically inspected by the Care Quality Commission [52]. Our training will include reminders about safe handling of specimens and will recommend revision of infection control measures, where needed.

## Results

### Surveillance Network Development

The COVID-19 pandemic has led to considerable numbers of practices joining the Oxford–RCGP RSC network. The collaboration with EMIS (Egton Medical Information Systems) Health has made it much easier for practices to contribute pseudonymized patient data at rapid speed and at a large scale. The network has tripled in size since January 2020, from 500 practices to 1724. Of these practices, 1076 are using the new ORCHID platform being developed in collaboration with EMIS for direct data extract. Pseudonymized data from over 13 million patients has enabled the growth of a rich database from which researchers are developing a deeper understanding of the COVID-19 pandemic and other diseases in general practice. We have opened a Facebook page and Twitter account to provide more online information and help (see Textbox 3).

### Virological Surveillance and Serosurveillance

We formerly had 100 practices taking part in the national influenza surveillance scheme. This has expanded to 322 for COVID-19 surveillance. As of August 17, 2020, the surveillance system has collected 5357 swabs and detected 581 cases of COVID-19 through extensive virological sampling. A total of 273 practices have contributed toward serosurveillance, collecting a baseline of 2000 samples and an additional 1000 samples per week. A total of 19,761 samples have been collected for serology as of August 17, 2020. We are also piloting serosurveillance in two cities in the northwest of England with a high incidence of COVID-19.

### Monitoring Vaccine Uptake and Effectiveness

We started collecting vaccine brands and batch numbers via our online portal on September 1, 2020, for the upcoming influenza season. We have made provisions in our dashboards and observatories for the changed vaccination policy for influenza and for virology sampling in the coming season. We have successfully piloted the secure transfer of data between ORCHID and PHE using the OxFile – Large File Exchange Service secure data transfer system [53].

### Supporting Priority Public Health Research

We have recruited 517 practices into the PRINCIPLE trial. A total of 440 patients have had successful screening and have been randomized. As of August 13, 2020, 537 patients have been recruited via general practitioner practices set up as official recruiting sites. Oxford–RCGP RSC practices have recruited 81.9% of these patients.

### Data Curation

Our key focus is the development of ORCHID and accessibility of our extended data set. Currently, 1076 practices use the ORCHID virtual server.

### Governance

We will put in place processes to allow approved researchers access to ORCHID.

## Discussion

### Principal Findings

We have tripled the sign-up to the Oxford–RCGP RSC and are well on our way to operationalizing this new extended network ahead of the coming winter, in a season complicated by SARS-CoV-2 circulation and an extended flu vaccination program.

The Oxford–RCGP RSC now has in place the most comprehensive set of linked data in its history, supplemented by reference lab virology and serology. Over half of our network will have data available on a daily basis via unprecedented, timely, remote researcher access to our themed data sets.

We have also introduced self-swabbing to increase safety and convenience for patients and practices, to enable direct relay of COVID-19 results to patients, and to reduce the burden of form filling. We have also extended the number and sophistication of our dashboards and observatories, adding interactivity and more extensive help.

Safety of practices remains our primary concern and we are not aware of any increased risk to practice staff or other patients from involvement in surveillance. Personal protective equipment was difficult to source at the start of the pandemic, as were SARS-CoV-2 tests. Both are now much more available to primary care.

### Comparison With Previous Work

Pandemic preparedness is part of the role of the RCGP RSC. The RCGP RSC has operated for over 50 years and has been involved in collecting samples to monitor disease and vaccine effectiveness through the Hong Kong flu pandemic of 1968-1969, the Russian flu of 1977-1978, the 2009 swine flu pandemic [54,55], and the first wave of the COVID-19 pandemic earlier this year.

The introduction of self-swabbing kits this year will enable wider distribution, allowing for an increase in the number of people to be tested. People in self-isolation and quarantine can access testing kits without leaving the confines of their homes, negating the risk of transmission to others with whom they interact with in transit [6]. Self-swabbing appears to be reliable [56,57].

We have shown considerable adaptability so far this year, tackling a wide range of issues and producing publicly available dashboards as well as research outputs [6-8,10,22].

### Strengths and Limitations

The strengths of our network lie in the willingness of practices to share data and the quality of those data. The Oxford–RCGP RSC team has a deep understanding of primary care data, the context of data recording, and the ability to process routine data. We have developed even closer relationships with computerized medical records system suppliers, especially EMIS. We have a long-term partnership with PHE and its predecessor bodies. There is a good understanding of the strengths and limitations of routine data and the capabilities of primary care.

The limitations of our system are those of all routine data, in that the data may not be complete or accurate. Ensuring we have good data quality is key for the reliability of our screening. There are few available comparators, but we know the network detects more new cases of some cardiovascular diseases than others [58]. Given our ambitious program of scaling, via a new pathway for virology surveillance based on patient-completed data, a further limitation is that these data are currently not linked back into their general practice record. We have also found that the success of open access testing means that individuals can be tested more quickly at a public testing station [59] than self-swabbing via a test through the surveillance system, thereby restricting the extent of possible collection.

While our network is large enough to report the relative incidence of common AEIs following influenza vaccination, comparing brands and vaccine types, it may be too small to comment on very rare events. Little is known about the anticipated AEIs with the new COVID-19 vaccine, so it is currently unclear what role we could play.

While we are part of many European and international networks, we did not have the opportunity to rapidly produce a shared international protocol. However, this document will include the learning from our decades of joint surveillance. It is being made available as a preprint to enable comments to be made and to allow learning to be shared.

### Conclusions

The Oxford–RCGP RSC has had a groundswell of interest in membership during the first wave of the COVID-19 pandemic. We have grown in network size and functionality. Our challenges are to maintain data quality and to help support our practices in identifying sufficient volunteers to deliver the required number of samples as we take on more general practice members. Another challenge is the uncertainty of a winter season with cocirculating COVID-19. The pace of change in our understanding of COVID-19 requires us to move ahead rapidly, rather than in close coordination with others. This protocol sets out our approach to surveillance in the coming season and makes our approach widely available for comment or use by others involved in surveillance.

## Appendix

Multimedia Appendix 1 In-house developed Systematized Nomenclature of Medicine Clinical Terms (SNOMED CT) coding tools to permit development of variable lists that match coding in computerized medical record (CMR) systems.

Multimedia Appendix 2 Newly released Systematized Nomenclature of Medicine Clinical Terms (SNOMED CT) concepts related to COVID-19 and SARS-CoV-2 grouped into relevant categories.

Multimedia Appendix 3 Flowchart of the 2020-2021 influenza season surveillance.

Multimedia Appendix 4 Royal College of General Practitioners (RCGP) Research and Surveillance Centre (RSC)–Public Health England (PHE) Joint Virology Summary, including use of virology data, July 2020.

## Abbreviations

AEI: adverse event of interest
DECISION-COVID: DEfining the CharacterIStIcs Of Individuals with suspected Novel COronaVIrus Disease and risk factors for development of the disease
DRIVE: Development of Robust and Innovative Vaccine Effectiveness
EMIS: Egton Medical Information Systems
FAIR: Findable, Accessible, Interoperable, and Reusable
HOCO: household contacts of confirmed COVID-19 cases
ILI: influenza-like illness
I-MOVE: Influenza–Monitoring Vaccine Effectiveness in Europe
MAINROUTE: Monitoring Attendance, INvestigation, Referral, and OUTcomEs in primary care: impact of and recovery from COVID-19 lockdown
NHS: National Health Service
ORCHID: Oxford–Royal College of General Practitioners Clinical Informatics Digital Hub
PHE: Public Health England
PRINCIPLE: Platform Randomised trial of INterventions against COVID-19 In older peoPLE
RAPTOR-C19: RAPid community Testing fOR COVID-19
RECAP: Remote COVID-19 Assessment in Primary Care
RCGP: Royal College of General Practitioners
RSC: Research and Surveillance Centre
SNOMED CT: Systematized Nomenclature of Medicine Clinical Terms
