# Supplementary material for: Influenza and Respiratory Virus Surveillance, Vaccine Uptake, and Effectiveness at a Time of Cocirculating COVID-19: Protocol for the English Primary Care Sentinel System for 2020-2021
Source: JMIR Public Health Surveill. 2021 Feb 19;7(2):e24341. doi: 10.2196/24341 (PMC7899204; doi:10.2196/24341)

**Multimedia Appendix 1. In-house developed SNOMED CT coding tools to permit development of variable lists which match to coding in CMR systems.**

These tools develop code lists in their Expression Constraint Language (ECL) format. This is the formal syntax of SNOMED CT and permits recreation of any code list knowing the relative contribution of various supertypes and subtypes in SNOMED CT.


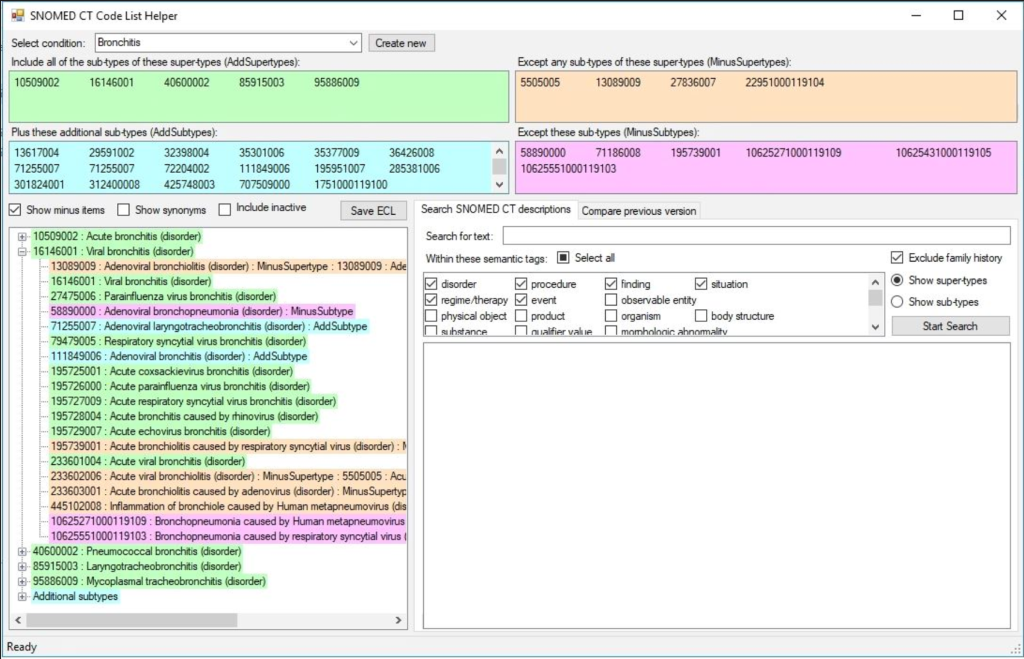

Supplement: Multimedia Appendix 1 [file publichealth_v7i2e24341_app1.docx]
