# Supplementary material for: Influenza and Respiratory Virus Surveillance, Vaccine Uptake, and Effectiveness at a Time of Cocirculating COVID-19: Protocol for the English Primary Care Sentinel System for 2020-2021
Source: JMIR Public Health Surveill. 2021 Feb 19;7(2):e24341. doi: 10.2196/24341 (PMC7899204; doi:10.2196/24341)
Supplement: Multimedia Appendix 2 [file publichealth_v7i2e24341_app2.docx]

**Multimedia Appendix 2. New release SNOMED-CT concepts related to COVID-19 and SARS-CoV-2 grouped into relevant categories.**

| **Status** | **Semantic tag** | **SNOMED CT Concept ID** | **Term** |  | |  |  |  |  |  |
| --- | --- | --- | --- | --- | --- | --- | --- | --- | --- | --- |
| **Lab-confirmed case** |  |  |  | | | | | |  |  |
|  | Clinical finding | 1240581000000104 | SARS-CoV-2 (severe acute respiratory syndrome coronavirus 2) detected | | | | | |  |  |
|  | Situation | 1300721000000109 | COVID-19 confirmed by laboratory test | | |  |  |  |  |  |
|  | Substance | 1240391000000107 | Antigen of severe acute respiratory syndrome coronavirus 2 | | | | |  |  |  |
|  | Substance | 1240411000000107 | Ribonucleic acid of severe acute respiratory syndrome coronavirus 2 | | | | | |  |  |
|  |  |  |  |  |  | |  |  |  |  |
| **Clinically-confirmed case** |  |  |  |  |  | |  |  |  |  |
|  | Disorder | 1240751000000100 | COVID-19 |  |  | |  |  |  |  |
|  | Situation | 1300731000000106 | COVID-19 confirmed using clinical diagnostic criteria | | | |  |  |  |  |
|  | Disorder | 1240561000000108 | Encephalopathy caused by SARS-CoV-2 (severe acute respiratory syndrome coronavirus 2) | | | | | | |  |
|  | Disorder | 1240571000000101 | Gastroenteritis caused by severe acute respiratory syndrome coronavirus 2 (disorder) | | | | | | |  |
|  | Disorder | 1240531000000103 | Myocarditis caused by SARS-CoV-2 (severe acute respiratory syndrome coronavirus 2) | | | | | | |  |
|  | Disorder | 1240521000000100 | Otitis media caused by SARS-CoV-2 (severe acute respiratory syndrome coronavirus 2) | | | | | | |  |
|  | Disorder | 1240551000000105 | Pneumonia caused by SARS-CoV-2 (severe acute respiratory syndrome coronavirus 2) | | | | | | |  |
|  | Disorder | 1240541000000107 | Infection of upper respiratory tract caused by severe acute respiratory syndrome coronavirus 2 (disorder) | | | | | | | |
|  | Disorder | 186747009 | Coronavirus infection |  |  | |  |  |  |  |
|  |  |  |  |  |  | |  |  |  |  |

**N.B. This list is not exhaustive. Further COVID-19 related coding materials and information provided by NHS Digital can be found here:** <https://hscic.kahootz.com/connect.ti/COVID19_info_sharing/grouphome>

| **Status** | **Semantic tag** | **SNOMED CT Concept ID** | **Term** |  |  |  |  |  |  |
| --- | --- | --- | --- | --- | --- | --- | --- | --- | --- |
| **Suspected** | Situation | 1240761000000102 | Suspected COVID-19 |  |  |  |  |  |  |
|  | Procedure | 1240451000000106 | Telephone consultation for suspected severe acute respiratory syndrome coronavirus 2 | | | | | |  |
| **Exposure** | Situation | 700217006 | Suspected coronavirus infection | |  |  |  |  |  |
|  | Event | 1240431000000104 | Exposure to SARS-CoV-2 (severe acute respiratory syndrome coronavirus 2) infection | | | | | |  |
| **Investigation** | Event | 1240441000000108 | Close exposure to SARS-CoV-2 (severe acute respiratory syndrome coronavirus 2) infection | | | | | |  |
|  | Procedure | 1240471000000102 | Measurement of severe acute respiratory syndrome coronavirus 2 antigen | | | | |  |  |
|  | Procedure | 1240511000000106 | Detection of SARS-CoV-2 (severe acute respiratory syndrome coronavirus 2) using polymerase chain reaction technique | | | | | | |
|  | Procedure | 1320971000000102 | Taking of swab for SARS-CoV-2 (severe acute respiratory syndrome coronavirus 2) | | | | | |  |
|  | Situation | 1321031000000105 | Self-taken swab for SARS-CoV-2 (severe acute respiratory syndrome coronavirus 2) completed | | | | | |  |
|  | Situation | 1321041000000101 | Self-taken swab for SARS-CoV-2 (severe acute respiratory syndrome coronavirus 2) offered | | | | | |  |
|  | Situation | 1321051000000103 | Swab for SARS-CoV-2 (severe acute respiratory syndrome coronavirus 2) taken by healthcare professional | | | | | | |
|  | Procedure | 1240461000000109 | Measurement of SARS-CoV-2 (severe acute respiratory syndrome coronavirus 2) antibody | | | | | |  |
|  | Observable entity | 1008541000000105 | Coronavirus ribonucleic acid detection assay | | |  |  |  |  |
|  | Observable entity | 1029481000000103 | Coronavirus nucleic acid detection assay | |  |  |  |  |  |
|  | Substance | 1240401000000105 | Antibody to severe acute respiratory syndrome coronavirus 2 | | | |  |  |  |
|  | Qualifier value | 1240421000000101 | Serotype severe acute respiratory syndrome coronavirus 2 | | | |  |  |  |
|  | Procedure | 1240461000000109 | Measurement of severe acute respiratory syndrome coronavirus 2 antibody | | | | |  |  |
|  |  |  |  |  |  |  |  |  |  |
| **Excluded** |  |  |  | | | | |  |  |
|  | Clinical finding | 1240591000000102 | SARS-CoV-2 (severe acute respiratory syndrome coronavirus 2) not detected | | | | |  |  |
|  | Situation | 1321111000000101 | COVID-19 excluded by laboratory test | |  |  |  |  |  |
|  | Situation | 1321101000000103 | COVID-19 excluded |  |  |  |  |  |  |
|  | Situation | 1321121000000107 | COVID-19 excluded using clinical diagnostic criteria | | |  |  |  |  |
