# Supplementary figures and images for: Influenza and Respiratory Virus Surveillance, Vaccine Uptake, and Effectiveness at a Time of Cocirculating COVID-19: Protocol for the English Primary Care Sentinel System for 2020-2021
Source: JMIR Public Health Surveill. 2021 Feb 19;7(2):e24341. doi: 10.2196/24341 (PMC7899204; doi:10.2196/24341)

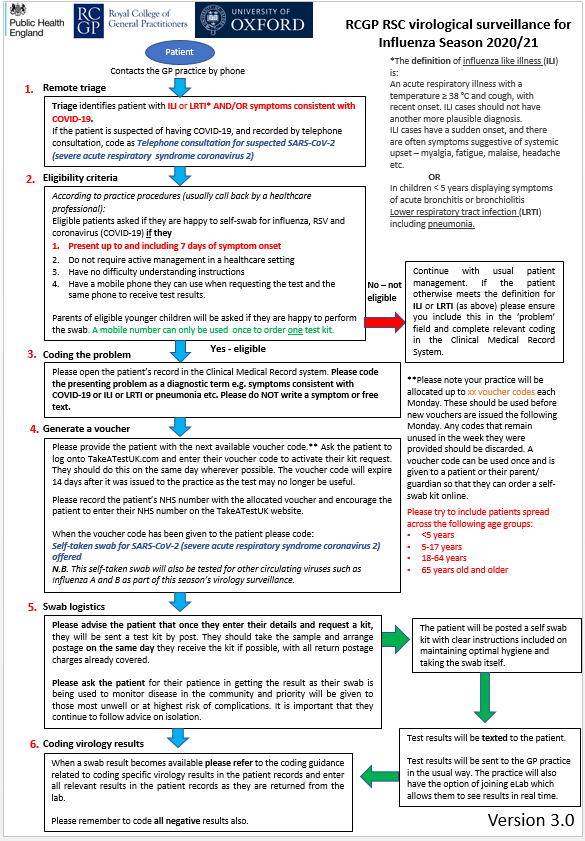

Supplement: Multimedia Appendix 3 [file publichealth_v7i2e24341_app3.docx]
