# Supplementary material for: Influenza and Respiratory Virus Surveillance, Vaccine Uptake, and Effectiveness at a Time of Cocirculating COVID-19: Protocol for the English Primary Care Sentinel System for 2020-2021
Source: JMIR Public Health Surveill. 2021 Feb 19;7(2):e24341. doi: 10.2196/24341 (PMC7899204; doi:10.2196/24341)
Supplement: Multimedia Appendix 4 [file publichealth_v7i2e24341_app4.docx]

# RCGP RSC/PHE Joint Virology Summary including use of virology data – July 2020

**RCGP/PHE Joint Virology Summary - July 2020**

| **Status** | **Clinical Description** | | **Sample type** | | **Sample collection type** | **Molecular Target** | | **Estimated no. samples expected** | | **PHE MOLIS Study Code** |
| --- | --- | --- | --- | --- | --- | --- | --- | --- | --- | --- |
| **Surveillance** | RCGP/PHE Enhanced surveillance.  300 practices currently | | Nasal swabs | | - Patients provide swab at GP practice or self-swab at home - Samples arrive by post | Currently COVID-19  Flu/RSV in flu season | | Currently 300/week  Flu season 1000/week | | **ERCGP19** |
| **Surveillance** | Saving Lives pilot study. RCGP/PHE Enhanced surveillance.  20 practices currently | | Nasal swabs | | - Patients self-swab at home - Samples arrive by post | Currently COVID-19  Flu/RSV in flu season | | Currently ~25/week | | **ECOVSL** |
| **Surveillance** | ERHOCO study  (Household contact) | | Nasal swab, oral fluid (OF) and bloods | | - Patients self-swab at home - Nasal swabs arrive by post - Convalescent OF and blood samples sent to Porton Down | Currently COVID-19 | | Currently ~150/week | | **ERHOCO** |
| **Trial of early interventions in COVID-19** | PRINCIPLE Trial  Community based therapy study  Patients provide swab on day 0  700 practices | | Nasal swabs | | - Patients self-swab at home - Samples arrive by post - Whole blood sample for serology | Currently COVID-19 | | Currently ~100/week and for next 6 weeks  Increase going forward, to have 2400 in total by March 2021 | | **ECOVP (was ERCGP19)** |
| **Rapid community COVID-19 test evaluation study** | RAPTOR C-19 - primary care setting for CONDOR (national diagnostic research and evaluation platform). Evaluation of rapid point-of-care tests against laboratory reference standard. Expanding community rapid test capacity (comparison of rapid molecular and serological tests vs reference tests in Colindale) for COVID-19. 110 practices have expressed interest, 40 sites in set-up. | | Point-of-care tests e.g. pharyngeal swabs, saliva, finger-prick blood, venous blood. | | - Point-of-care tests in general practice setting. - Nasal swabs as reference standard to Colindale - Serology samples as reference standar – testing centre TBC | COVID-19 | | Sample size expected to be similar to Principle study but dependent on prevalence. Estimated 1000-1500 participants per point-of-care test. | | **ECOVR1** |
| **Disease Burden** | RSV ComNetII project  Disease burden of RSV in <5 year olds in primary care | | Nasal swabs | | - GP practice - Samples arrive by post | RSV | | Estimated ~400 additional virology swabs over the winter | | **Not yet defined** |
| **Point of Care Testing (POCT); VE study** | | DRIVE 12 RCGP RSC practices enrolled in Point Of Care Testing (POCT) study; vaccine effectiveness | | Nasal swabs | - Patients presenting with fever recruited to provide nasal swab at GP practice | | Influenza A and B (during flu season) | | ~120 samples/ week |  |
| **Surveillance; VE study** | | IMOVE+  Specimens are collected from ILI cases ≥65 years who consult their GP within seven days of symptom onset; to collect epidemiological and clinical information on patients with COVID-19 as well as virological information on SARS-CoV-2 | | Nasal swabs | - ILI cases ≥65 years who consult their GP within seven days of symptom onset | | - subtype-specific laboratory-confirmed influenza A - laboratory-confirmed influenza B overall and if available by lineage (B Victoria/B Yamagata) - laboratory-confirmed influenza by clade (where possible) | | No additional samples |  |
|  | | DECISION  To develop a clinical prediction model for COVID-19 and subsequent complications (e.g. hospitalisation, admission to an intensive care unit, death, etc) which can be used to better target future shielding strategies, social distancing measures and vaccination programmes | |  |  | |  | | No additional samples |  |
| **Data analytics** | | MAINROUTE  describe and analyse the impact of the COVID-19 lockdown on presentation patterns, diagnoses, monitoring and outcomes of common non-communicable diseases | | ORCHID-Surveillance platform (practice-level aggregated data); ORCHID-Epi platform | - Descriptive dashboard data analytics - Time series analyses - Long-term outcomes of changes in consultation patterns in primary care | | - All-cause mortality - Cause-specific mortality (for each specified condition) - Hospital admission | | No additional samples |  |
| **Metadata / Curation / Common data model (CDM)** | | EHDEN project  Supports implementation of the common data model that allows RCGP RSC to participate in a wider range of international collaborating projects; Standardising >100 million patient records to the OMOP-CDM | |  | - Phase 1 - ETL design and infrastructure setup - Phase 2 - Operationalisation of OHDSI systems/ tools - Phase 3- Integration RCGP RSC with EHDEN ecosystem | |  | | No additional samples |  |
| **Curation / Unique longitudinal data resource** | | Wellcome QQG  Creating a longitudinal linked sentinel database of over 50  years clinical and virology data and prospective research platform | | Linking all historic virology data to RCGP RSC practice data. | All data. The project also funds a virologist who will work with PHE to reliably curate these data. | |  | | No additional samples |  |
| **Enhanced remote diagnosis of COVID-19** | | RECAP  Validation of an early warning score for use in GP-patient consultations mainly by phone or video, in the context of COVID-19. | | Linking COVID-19 data from GPs electronic health record with outcome data from acute trusts on admission, COVID-19 status, ICU admission and death |  | |  | | No additional samples |  |
